# Supplementary material for: Precancerous liver diseases do not cause increased mutagenesis in liver stem cells
Source: Commun Biol. 2021 Nov 18;4:1301. doi: 10.1038/s42003-021-02839-y (PMC8602268; doi:10.1038/s42003-021-02839-y)
Supplement: Supplementary file 2 — Supplementary Information [file 42003_2021_2839_MOESM2_ESM.pdf]

Supplementary information

Precancerous liver diseases do not cause increased mutagenesis in liver stem cells

Luan Nguyen[1,$], Myrthe Jager[1,$], Ruby Lieshout[2], Petra E. de Ruiter[2], Mauro D. Locati[1], Nicolle Besselink[1], Bastiaan van der Roest[1], Roel Janssen[1], Sander Boymans[1], Jeroen de Jonge[2], Jan N.M. IJzermans[2], Michail Doukas[2], Monique M.A. Verstegen[2], Ruben van Boxtel[3], Luc J.W. van der Laan[2], Edwin Cuppen[1,4,*], Ewart Kuijk[1,*]

[1] Center for Molecular Medicine and Oncode Institute, University Medical Center Utrecht, Utrecht, The Netherlands
[2] Erasmus Medical Center, Rotterdam, The Netherlands
[3] Princess Máxima Center, Utrecht, The Netherlands
[4] Hartwig Medical Foundation, Amsterdam, The Netherlands

[$] These authors contributed equally to this work
[*] Corresponding authors

Supplementary note 1: Power analysis

We performed a power analysis to assess the minimum detectable changes to mutation accumulation and mutational signature contributions given our number of samples. We used the *pwr* R package to calculate hypothetical detectable effect sizes using a significance level of 0.05 and a power of 0.8. We used the *pwr.t2n.test()* and *pwr.chisq.test()* functions to calculate Cohen's D and Cohen's W respectively.

Given that we have 8 healthy patients, 5 with alcoholic cirrhosis, 5 with NASH and 3 with PSC, we expect to be able to detect an effect size (Cohen's D) from 1.51 to 1.83 for mutation accumulation (i.e. as shown in **Figure 2**). For changes in SBS contributions (i.e. as shown in **Figure 3**), the degrees of freedom (DF) is 9 (10−1 signatures x 2−1 conditions), and thus we expect to detect an effect size (Cohen's W) of 1.10 to 1.19 when the total number of samples is 13 or 11 respectively. For changes in indel contributions, the degrees of freedom (DF) is 6 (7−1 signatures x 2−1 conditions), and thus we expect to detect an effect size (Cohen's W) of 1.02 to 1.11 when the total number of samples is 13 or 11 respectively.

When then compared this with similar experimental setups in published studies to determine what these hypothetical effect sizes signify. Kuijk *et al.* [27] found that organoids cultured in 20% oxygen (n=5) accumulated ~1.5 times more SBSs than those cultured in 3% oxygen (n=3) (p=0.003, two-sided t-test). Here, the detectable effect size (Cohen's D) would be 2.06. Organoids cultured in 20% oxygen also showed higher relative contribution of SBS5 (~30%) than those cultured in 3% oxygen (~10%). In this comparison, the DF) was 6 = 7−1 signatures x 2−1 conditions) and the total number of samples was 8, thus yielding a detectable effect size (Cohen's W) of 1.31. Drost *et al.* [47] found that MLH1 knockout organoids (n=3) accumulated more SBSs (~25) and indels (~100) compared to wild type organoids (n=4) (~5 for both SBSs and indels). Here, the detectable effect size (Cohen's D) would be 2.22. Jager *et al.* [48] found that organoids derived from ERCC1 knockout mouse livers (n=3) showed increased relative contribution of SBS8 (~60%) compared to those derived from wild type mice (n=3) (~25%). In this comparison, the DF was 9 (= 10−1 signatures x 2−1 conditions) and the total number of samples was 3, thus yielding a detectable effect size (Cohen's W) of 1.91.

Thus, the sample sizes in our study would allow us to detect effect sizes similar to those found in the aforementioned published studies.

Supplementary figures

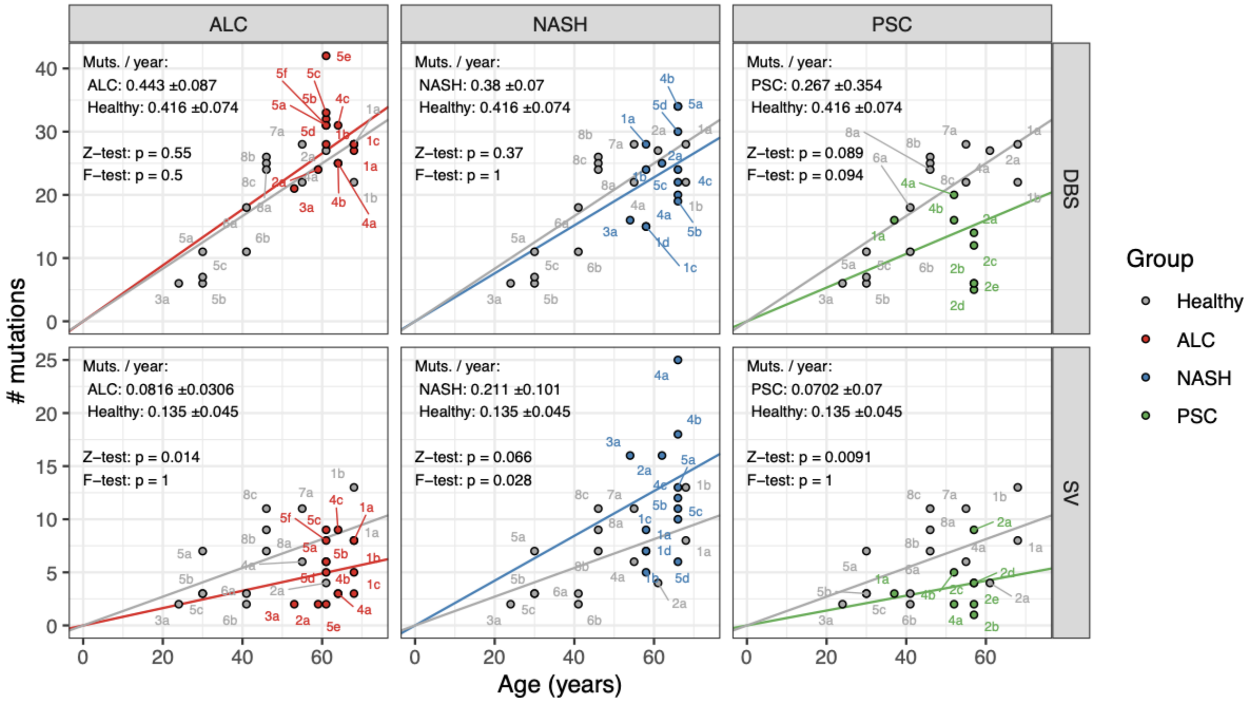

Supplementary figure 1: Accumulation of double base substitutions (DBS) and structural variants (SV) in organoids derived from biopsies of healthy livers compared to those from patients with diseased livers. ALC: alcoholic cirrhosis, NASH: non-alcoholic steatohepatitis, PSC: primary sclerosing cholangitis. Each point is labelled by patient number and clone letter. Two-sided Z-tests were performed to determine whether there was a significant difference between the linear mixed effects regressions (i.e. the rate of mutation accumulation) of the disease versus healthy ICOs. One-sided F-tests were performed to determine whether there was a significant increase in variance in rate of mutation accumulation in disease samples versus healthy samples. ± values indicate the 95% confidence interval range of each regression and 'p' indicates the p-values of the Z-tests and F-tests.

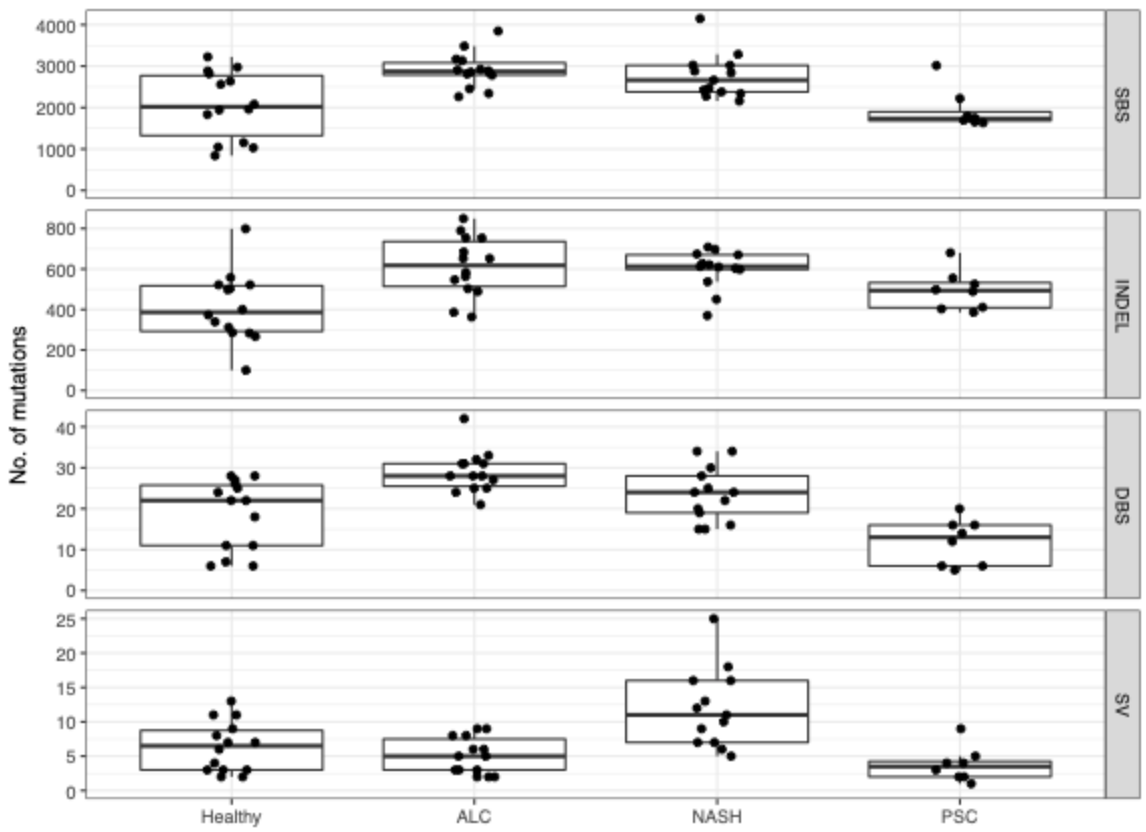

Supplementary figure 2: Mutational load per mutation type in organoids derived from biopsies of healthy livers, and livers from patients with alcoholic cirrhosis (ALC), non-alcoholic steatohepatitis (NASH), or primary sclerosing cholangitis (PSC). Each dot represents one organoid line. Boxplot boxes show the interquartile range (IQR) and whiskers show the largest/smallest values within 1.5 times the IQR. Each dot is one organoid clone.

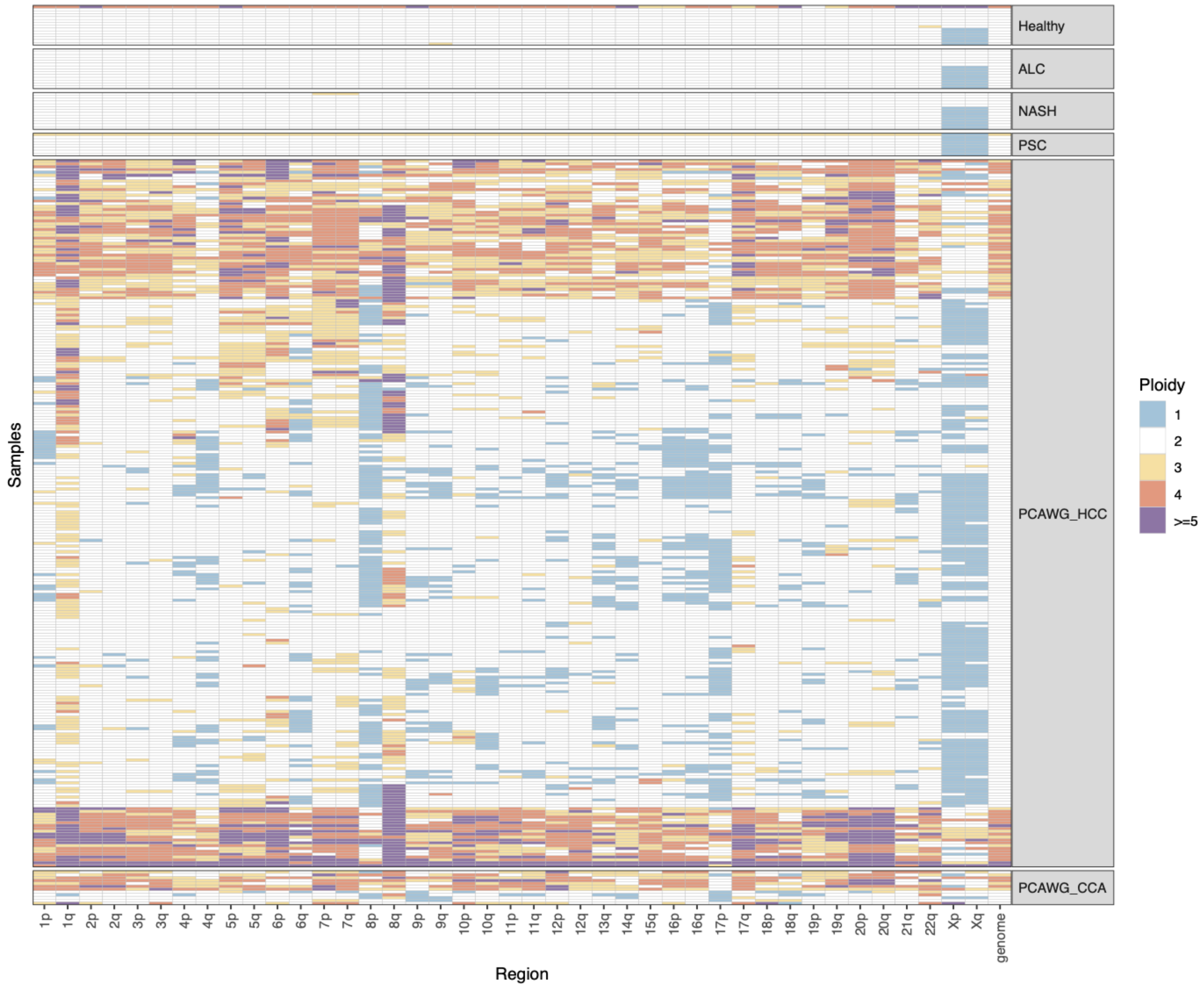

Supplementary figure 3: Chromosome arm copy number profiles in organoids derived from biopsies of healthy, diseased and cancerous livers. ALC: alcoholic cirrhosis, NASH: non-alcoholic steatohepatitis, PSC: primary sclerosing cholangitis, HCC: hepatocellular carcinoma, CCA: cholangiocarcinoma. For one 60-year-old patient with hepatocellular carcinoma biopsies from 5 locations were taken from the liver (HCC_multibiopsy). Profiles for HCC (PCAWG_HCC; n=248) and CCA (PCAWG_CCA; n=12) samples from the Pan-Cancer Analysis of Whole Genomes (PCAWG) consortium are also shown.

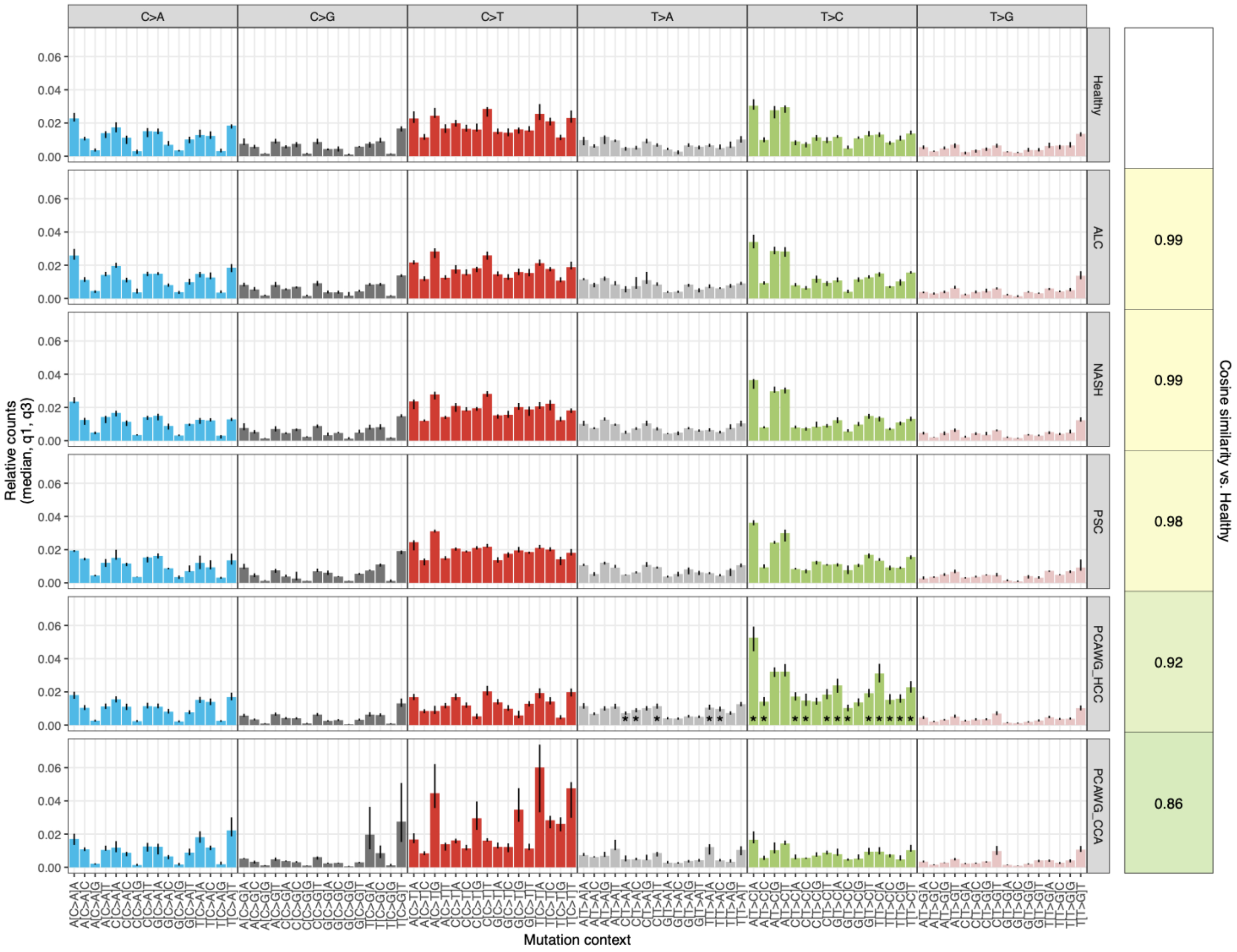

Supplementary figure 4: Trinucleotide substitution profiles in organoids derived from biopsies of healthy, diseased and cancerous livers. ALC: alcoholic cirrhosis, NASH: non-alcoholic steatohepatitis, PSC: primary sclerosing cholangitis, HCC: hepatocellular carcinoma, CCA: cholangiocarcinoma. Profiles for HCC (PCAWG_HCC; n=248) and CCA (PCAWG_CCA; n=12) samples from the Pan-Cancer Analysis of Whole Genomes (PCAWG) consortium are also shown. x-axis labels show the base substitution within the square brackets and the 5' and 3' flanking bases. Bars show the median relative mutation counts (i.e. normalized by total no. of mutations per sample), with error bars showing the 1st and 3rd quartiles. Asterisks indicate a significant increase in mutation context load in a disease/cancer sample versus healthy liver organoids (Wilcoxon rank sum test, Bonferroni adjusted p-value<0.01). Right of the bar plots, cosine similarities of the median profiles of the disease/cancer samples compared to that of the healthy liver organoids.

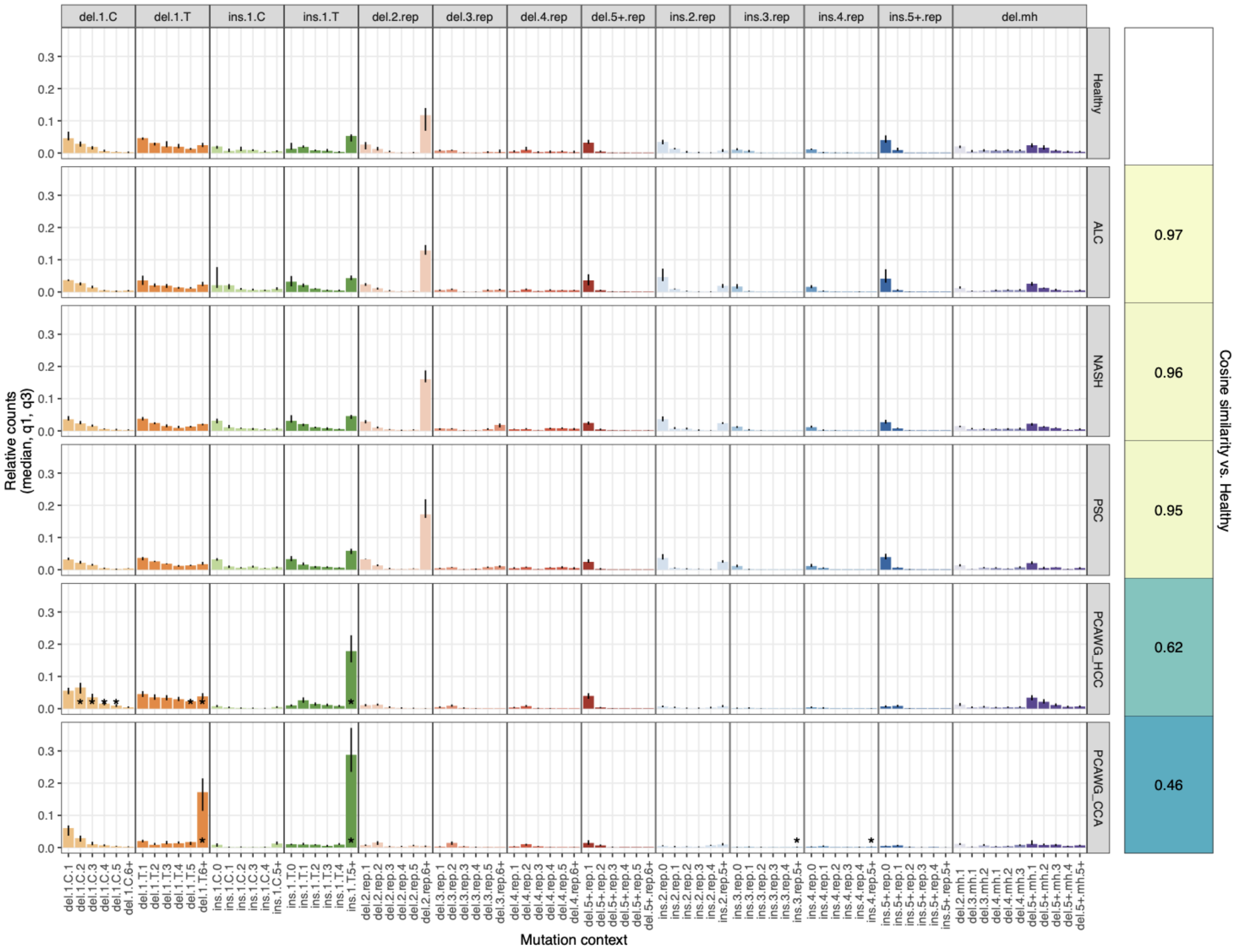

Supplementary figure 5: Indel profiles in organoids derived from biopsies of healthy, diseased and cancerous livers. ALC: alcoholic cirrhosis, NASH: non-alcoholic steatohepatitis, PSC: primary sclerosing cholangitis, HCC: hepatocellular carcinoma, CCA: cholangiocarcinoma. Profiles for HCC (PCAWG_HCC; n=248) and CCA (PCAWG_CCA; n=12) samples from the Pan-Cancer Analysis of Whole Genomes (PCAWG) consortium are also shown. x-axis labels are in the format (each separated by a dot): (i) deletion or insertion; (ii) no. of deleted/inserted bases; (iii) indel belongs in homopolymer repeats of C/T (C/T), multibase repeat region (rep), or has flanking microhomology (mh); (iv) no. of repeats of C/T, no. of repeat units, or number of homologous bases. Bars show the median relative mutation counts (i.e. normalized by total no. of mutations per sample), with error bars showing the 1st and 3rd quartiles. Asterisks indicate a significant increase in mutation context load in a disease/cancer sample versus healthy liver organoids (Wilcoxon rank sum test, Bonferroni adjusted p-value<0.01). Right of the bar plots, cosine similarities of the median profiles of the disease/cancer samples compared to that of the healthy liver organoids.

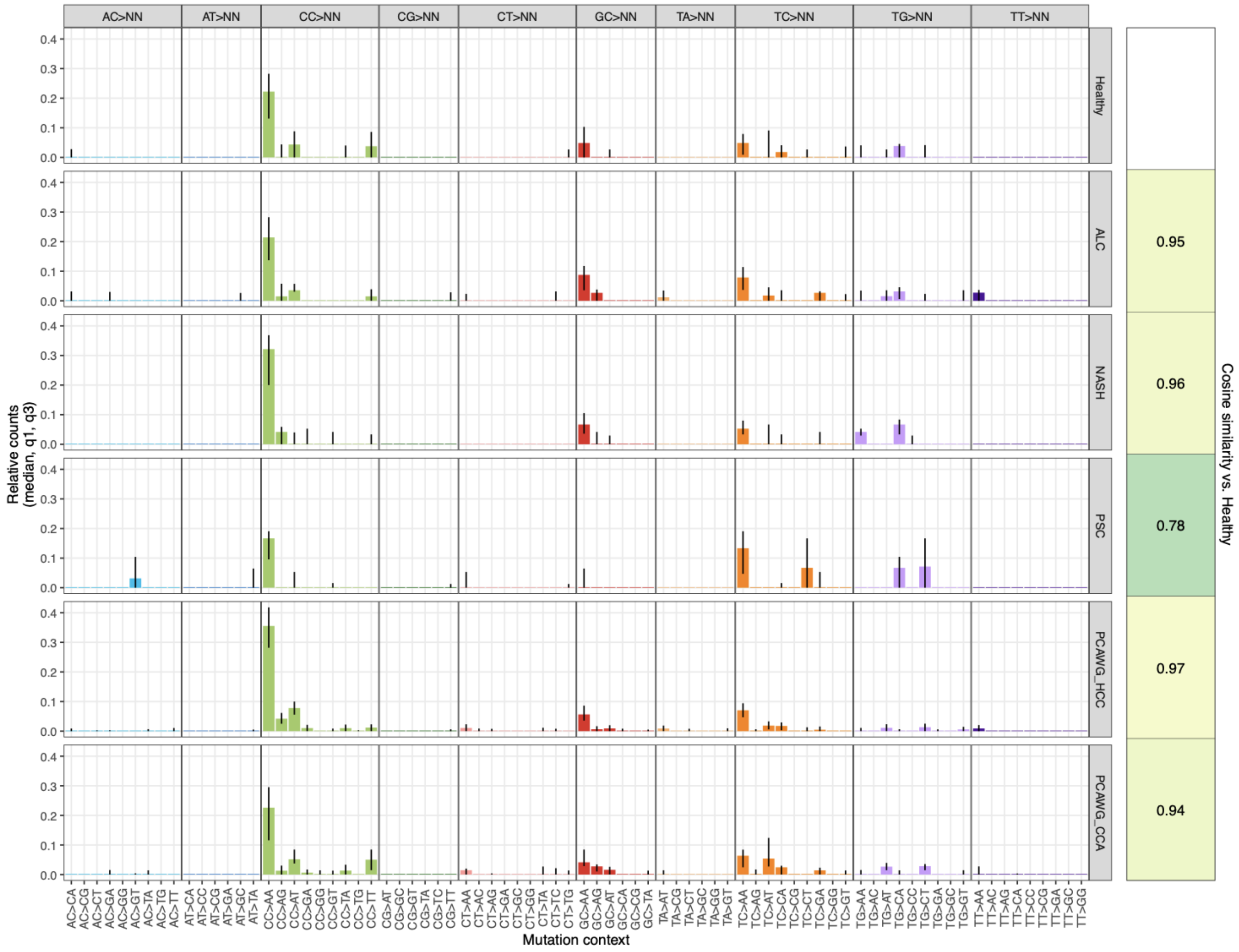

Supplementary figure 6: Double base substitution (DBS) profiles in organoids derived from biopsies of healthy, diseased and cancerous livers. ALC: alcoholic cirrhosis, NASH: non-alcoholic steatohepatitis, PSC: primary sclerosing cholangitis, HCC: hepatocellular carcinoma, CCA: cholangiocarcinoma. Profiles for HCC (PCAWG_HCC; n=248) and CCA (PCAWG_CCA; n=12) samples from the Pan-Cancer Analysis of Whole Genomes (PCAWG) consortium are also shown. x-axis labels indicate the dinucleotide change. Bars show the median relative mutation counts (i.e. normalized by total no. of mutations per sample), with error bars showing the 1st and 3rd quartiles. Asterisks indicate a significant increase in mutation context load in a disease/cancer sample versus healthy liver organoids (Wilcoxon rank sum test, Bonferroni adjusted p-value<0.01). Right of the bar plots, cosine similarities of the median profiles of the disease/cancer samples compared to that of the healthy liver organoids.

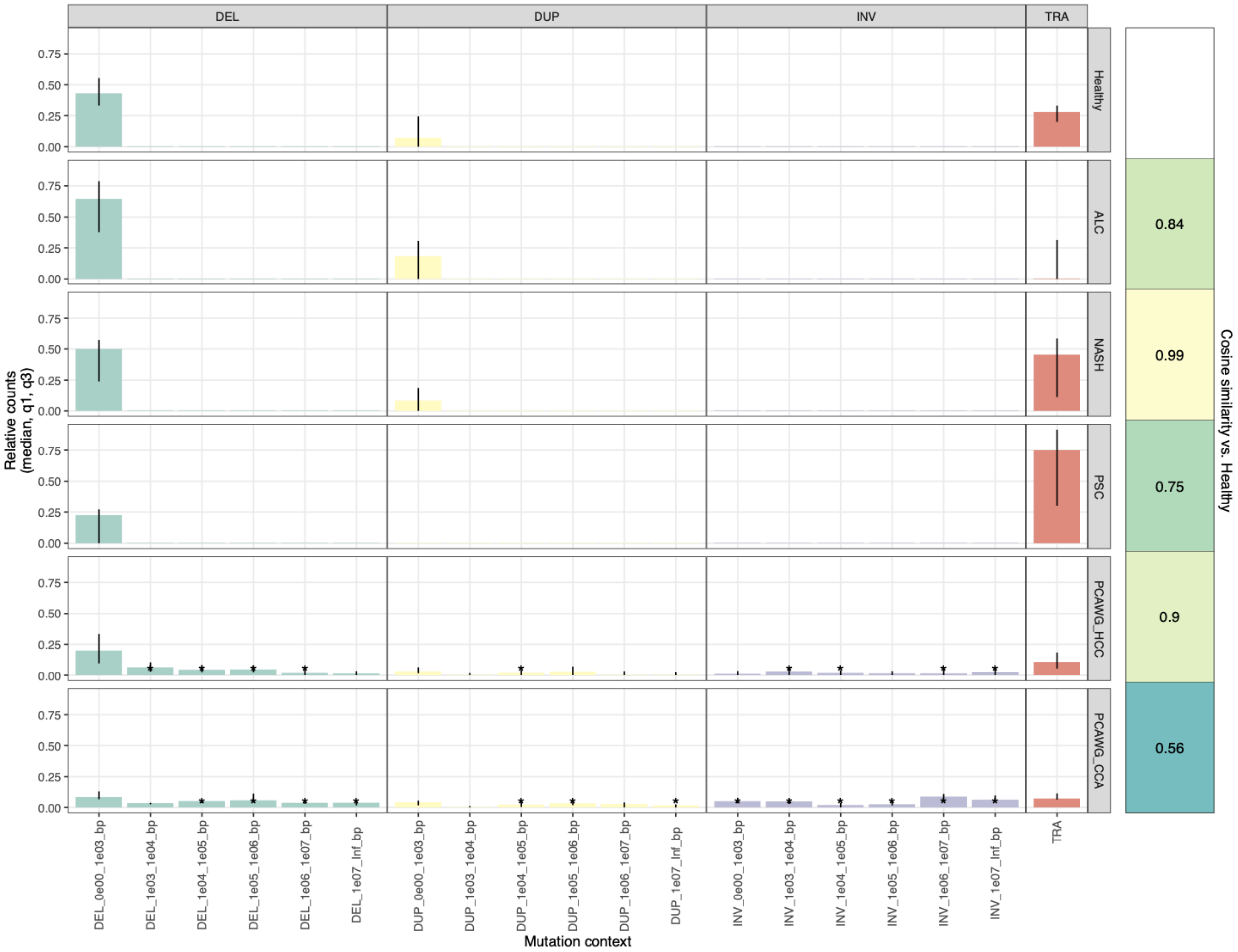

Supplementary figure 7: Structural variant (SV) profiles in organoids derived from biopsies of healthy, diseased and cancerous livers. ALC: alcoholic cirrhosis, NASH: non-alcoholic steatohepatitis, PSC: primary sclerosing cholangitis, HCC: hepatocellular carcinoma, CCA: cholangiocarcinoma. Profiles for HCC (PCAWG_HCC; n=248) and CCA (PCAWG_CCA; n=12) samples from the Pan-Cancer Analysis of Whole Genomes (PCAWG) consortium are also shown. x-axis labels indicate the SV stratified by mutation type and length interval. Bars show the median relative mutation counts (i.e. normalized by total no. of mutations per sample), with error bars showing the 1st and 3rd quartiles. Asterisks indicate a significant increase in mutation context load in a disease/cancer sample versus healthy liver organoids (Wilcoxon rank sum test, Bonferroni adjusted p-value<0.01). Right of the bar plots, cosine similarities of the median profiles of the disease/cancer samples compared to that of the healthy liver organoids.
