# Supplementary material for: Precancerous liver diseases do not cause increased mutagenesis in liver stem cells
Source: Commun Biol. 2021 Nov 18;4:1301. doi: 10.1038/s42003-021-02839-y (PMC8602268; doi:10.1038/s42003-021-02839-y)
Supplement: Supplementary file 3 — Description of Additional Supplementary Files [file 42003_2021_2839_MOESM3_ESM.pdf]

Description of Additional Supplementary Files

File name: Supplementary Data 1

Description: Sample metadata.

File name: Supplementary data 2

Description: Genes enriched in non-synonymous mutations as determined by dndscv.

File name: Supplementary data 3

Description: Mutation context and absolute mutational signature contributions per sample.

File name: Supplementary data 4

Description: Shapiro test on the mutational load per mutation type per disease status group. Since for some patients multiple intrahepatic cholangiocyte organoid (ICO) clones were derived, the mean mutational load over the ICO clones for each patient was first calculated before performing the Shapiro test.
