# Supplementary material for: Precancerous liver diseases do not cause increased mutagenesis in liver stem cells
Source: Commun Biol. 2021 Nov 18;4:1301. doi: 10.1038/s42003-021-02839-y (PMC8602268; doi:10.1038/s42003-021-02839-y)
Supplement: Supplementary file 8 — Reporting Summary [file 42003_2021_2839_MOESM8_ESM.pdf]

nature portfolio

Corresponding author(s): Ewart Kuijk

Last updated by author(s): Oct 14, 2021

Reporting Summary

Nature Portfolio wishes to improve the reproducibility of the work that we publish. This form provides structure for consistency and transparency in reporting. For further information on Nature Portfolio policies, see our Editorial Policies and the Editorial Policy Checklist.

Statistics

For all statistical analyses, confirm that the following items are present in the figure legend, table legend, main text, or Methods section.

n/a	Confirmed	
☐	☒	The exact sample size (*n*) for each experimental group/condition, given as a discrete number and unit of measurement
☐	☒	A statement on whether measurements were taken from distinct samples or whether the same sample was measured repeatedly
☐	☒	The statistical test(s) used AND whether they are one- or two-sided<br>*Only common tests should be described solely by name; describe more complex techniques in the Methods section.*
☒	☐	A description of all covariates tested
☐	☒	A description of any assumptions or corrections, such as tests of normality and adjustment for multiple comparisons
☐	☒	A full description of the statistical parameters including central tendency (e.g. means) or other basic estimates (e.g. regression coefficient) AND variation (e.g. standard deviation) or associated estimates of uncertainty (e.g. confidence intervals)
☐	☒	For null hypothesis testing, the test statistic (e.g. *F*, *t*, *r*) with confidence intervals, effect sizes, degrees of freedom and *P* value noted<br>*Give P values as exact values whenever suitable.*
☒	☐	For Bayesian analysis, information on the choice of priors and Markov chain Monte Carlo settings
☒	☐	For hierarchical and complex designs, identification of the appropriate level for tests and full reporting of outcomes
☒	☐	Estimates of effect sizes (e.g. Cohen's *d*, Pearson's *r*), indicating how they were calculated

Our web collection on statistics for biologists contains articles on many of the points above.

Software and code

Policy information about availability of computer code

Data collection	No code was used for data collection
Data analysis	The Hartwig Medical Foundation variant calling pipeline (https://github.com/hartwigmedical/pipeline; v4.8) was used for variant calling. All analyses in R were performed using v4.0.3. Extraction of mutation contexts and subsequent mutational signature analysis was performed using the mutSigExtractor R package (https://github.com/UMCUGenetics/mutSigExtractor; v1.23). The geneDriverAnnotator R package (https://github.com/UMCUGenetics/geneDriverAnnotator; v1.0) was used for annotation of mutation type and effect for each SNV/indel. The dndscv R package (v0.0.1.0) was used to identify genes that were enriched for non-synonymous mutations. The nlme R package (v3.1) was used to fit a linear mixed effects regressions.

For manuscripts utilizing custom algorithms or software that are central to the research but not yet described in published literature, software must be made available to editors and reviewers. We strongly encourage code deposition in a community repository (e.g. GitHub). See the Nature Portfolio guidelines for submitting code & software for further information.

Data

Policy information about availability of data

All manuscripts must include a data availability statement. This statement should provide the following information, where applicable:

- Accession codes, unique identifiers, or web links for publicly available datasets
- A description of any restrictions on data availability
- For clinical datasets or third party data, please ensure that the statement adheres to our policy

The BAM files from the whole-genome sequencing data generated in the current study are available at EGA (https://www.ebi.ac.uk/ega/home) under accession numbers EGAS00001002983 and EGAS00001005384. BAM files from hepatocellular carcinoma and cholangiocarcinoma patients from the Pan-Cancer Analysis

Whole Genomes (PCAWG) consortium were obtained under request number DACO-5333. For access to the PCAWG BAM files, researchers will need to request access via the ICGC Data Access Compliance Office (DACO; https://daco.icgc.org/). The VCF and tabular files produced from somatic variant calling are available at https://zenodo.org/record/5562381.

Field-specific reporting

Please select the one below that is the best fit for your research. If you are not sure, read the appropriate sections before making your selection.

[x] Life sciences   [ ] Behavioural & social sciences   [ ] Ecological, evolutionary & environmental sciences

For a reference copy of the document with all sections, see nature.com/documents/nr-reporting-summary-flat.pdf

Life sciences study design

All studies must disclose on these points even when the disclosure is negative.

Sample size	Sample sizes were determined by availability of liver material from liver transplantations and by the success rates to derive organoid cultures from the biopsies. We aimed to include at least 5 patients per disease condition to capture the variation between the patients. For the PSC patients, success rates to establish organoid cultures were too low to reach this number.
Data exclusions	No data was excluded from the analysis
Replication	Multiple clones per patient and multiple patients per disease condition were used as replicates for our findings
Randomization	Randomization was not performed due to the scarcity of the patient material
Blinding	Blinding was not relevant for our study because all analyses were performed bioinformatically using the same pipeline for all conditions

Reporting for specific materials, systems and methods

We require information from authors about some types of materials, experimental systems and methods used in many studies. Here, indicate whether each material, system or method listed is relevant to your study. If you are not sure if a list item applies to your research, read the appropriate section before selecting a response.

Materials & experimental systems

n/a	Involved in the study	
[x]	[ ]	Antibodies
[x]	[ ]	Eukaryotic cell lines
[x]	[ ]	Palaeontology and archaeology
[x]	[ ]	Animals and other organisms
[x]	[ ]	Human research participants
[x]	[ ]	Clinical data
[x]	[ ]	Dual use research of concern

Methods

n/a	Involved in the study	
[x]	[ ]	ChIP-seq
[x]	[ ]	Flow cytometry
[x]	[ ]	MRI-based neuroimaging
